# Δ1-Dehydrogenation and C20 Reduction of Cortisone and Hydrocortisone Catalyzed by *Rhodococcus* Strains

**DOI:** 10.3390/molecules25092192

**Published:** 2020-05-07

**Authors:** Stefania Costa, Federico Zappaterra, Daniela Summa, Bruno Semeraro, Giancarlo Fantin

**Affiliations:** 1 Department of Life Sciences and Biotechnology, University of Ferrara, Via L. Borsari, 46, 44121 Ferrara, Italy; federico.zappaterra@unife.it; 2 GATE SRL, Via L. Borsari, 46, 44121 Ferrara, Italy; daniela.summa@unife.it (D.S.); bsemeraro@gategreen.it (B.S.); 3 Department of Chemical and Pharmaceutical Sciences, University of Ferrara, Via L. Borsari, 46, 44121 Ferrara, Italy; giancarlo.fantin@unife.it

**Keywords:** cortisone, hydrocortisone, biotransformations, prednisone, prednisolone, 20β-hydroxy-prednisone, 20β-hydroxy-prednisolone, *Rhodococcus* spp.

## Abstract

Prednisone and prednisolone are steroids widely used as anti-inflammatory drugs. Development of the pharmaceutical industry is currently aimed at introducing biotechnological processes and replacing multiple-stage chemical syntheses. In this work we evaluated the ability of bacteria belonging to the *Rhodococcus* genus to biotransform substrates, such as cortisone and hydrocortisone, to obtain prednisone and prednisolone, respectively. These products are of great interest from a pharmaceutical point of view as they have higher anti-inflammatory activity than the starting substrates. After an initial lab-scale screening of 13 *Rhodococcus* strains, to select the highest producers of prednisone and prednisolone, we reported the 200 ml-batch scale-up to test the process efficiency and productivity of the most promising *Rhodococcus* strains. *R. ruber*, *R. globerulus* and *R. coprophilus* gave the Δ1-dehydrogenation products of cortisone and hydrocortisone (prednisone and prednisolone) in variable amounts. In these biotransformations, the formation of products with the reduced carbonyl group in position C_20_ of the lateral chain of the steroid nucleus was also observed (i.e., 20β-hydroxy-prednisone and 20β-hydroxy-prednisolone). The yields, the absence of collateral products, and in some cases the absence of starting products allow us to say that cortisone and hydrocortisone are partly degraded.

## 1. Introduction

Steroids are lipids belonging to the terpenes class, and from a chemical-structural point of view they contain a tetracyclic system of carbon atoms (cyclopentanoperhydrophenanthrene). This type of compound is widespread in nature: thousands of steroids have been identified in living systems. Over 250 sterols and related compounds have been reported to occur in plants (e.g., phytosterols, diosgenin, and brassinosteroids), insects (e.g., ecdysteroids), vertebrates (e.g., cholesterol; corticosteroids: glucocorticoids, mineralocorticoids; sex hormones: androgens, estrogens; bile acids, vitamin D; and neurosteroids), and lower eukaryotes: yeasts and fungi (e.g., ergosterol and ergosteroids) [1,2,3].

Steroid-based drugs present a broad range of therapeutic applications and represent the highest marketed category of pharmaceuticals, after antibiotics, with an annual production of more than one million tons. Currently, about 300 steroid drugs are known, and this number tends to grow. Their production represents the second category in the pharmaceutical market after antibiotics [4]. 

A large number of steroids are used as anti-inflammatory agents, immunosuppressants, progestational agents, diuretics, anabolics, and contraceptives [5,6,7]. Some steroid compounds are used for the treatment of prostate and breast cancer [8], as replacement therapy in patients with primary or secondary adrenal insufficiency, as adrenal suppression therapy in congenital adrenal hyperplasia and glucocorticoid resistance for adrenal insufficiency [9], for the prevention of heart disease [10], as antifungal agents [11], and as active ingredients useful for the treatment of obesity [12] and AIDS [13]. Recently, the antiviral activity against the herpes simplex virus type I of some steroid glycosides was determined [14].

The structure of steroids is closely related to their biological activity, i.e., the type, number, and regio and stereo position of the functional groups attached to the steroid core and the oxidation state of the rings. For example, the presence of an oxygen-containing functional group at C_11β_ is crucial for inflammatory activity, the hydroxyl group linked to C_17β_ determines the androgenic properties, the aromatization of ring A results in estrogenic effects, and the corticoids have a 3-oxo-5-ene moiety and the pregnane side chain at C_17_ [5,15]. 

In this field, corticosteroids are a group of hormones, produced by the adrenal gland cortex, belonging to the steroid class. They are used for their anti-inflammatory and immunosuppressive properties and for their effects on metabolism. They are divided into glucocorticoids, which control the metabolism of carbohydrates, lipids, and proteins, and mineralcorticoids, which control the electrolyte levels and the amount of water present in the blood.

**Figure 1:**
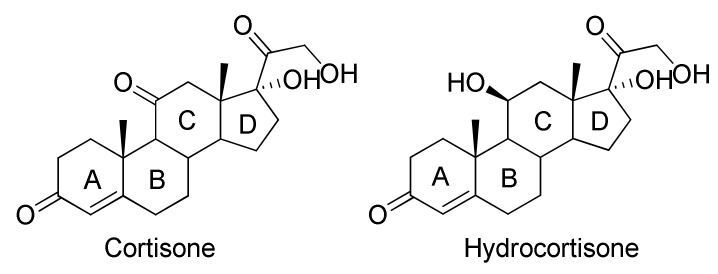
Chemical structures of cortisone and hydrocortisone.

Cortisone and cortisol (hydrocortisone) belong to the glucocorticoid class. They are characterized by the presence of a ketone group in position C_3_, a double bond in position C_4,_ and a chain COCH_2_OH in C_17_, and they differ from each other by the fact that in the C_11_ position cortisone has a carbonyl group while in the structure of hydrocortisone there is a hydroxyl group (Figure 1).

Cortisone and hydrocortisone have a well-known anti-inflammatory activity, and it is equally known that the presence of additional/second double bond at the C_1_-C_2_ position in prednisone and prednisolone increases the mentioned activity: in particular Δ1-hydrocortisone (prednisolone) acetate is four times more active than hydrocortisone acetate [16].

Therapeutic effects of steroids can often parallel undesirable side effects, especially when high doses and long-term therapy are required. For this reason the use of more powerful drugs, such as prednisone and prednisolone, can reduce these effects as a lower dose of drug is administered to obtain the same therapeutic effect; prednisone and prednisolone have in fact proved to be four to five times more active than cortisone and hydrocortisone [17].

**Figure 2:**
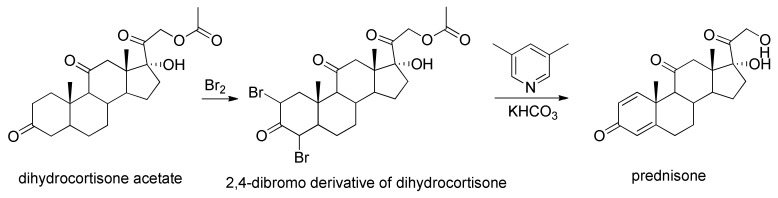
Synthesis of prednisone starting from dihydrocortisone acetate.

The synthesis of prednisone and prednisolone by chemical methods requires various reaction steps, in particular regarding prednisone as there are various ways of synthesizing it. In one of these, it is synthesized from dihydrocortisone acetate. In the given example, this compound undergoes dibromination by molecular bromine, giving a 2,4-dibromo derivative of dihydrocortisone. Dehydrobromination with 3,5-lutidine, followed by subsequent hydrolysis of the acetyl group using potassium bicarbonate, gives prednisone [18] (Figure 2).

**Figure 3:**
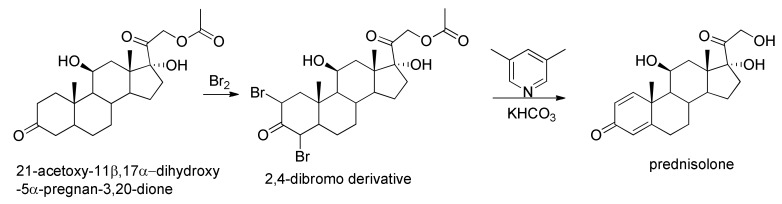
Synthesis of prednisolone starting from 21-acetoxy-11β,17α-dihydroxy-5α-pregnan-3,20-dione.

As for the chemical synthesis of prednisolone, one of various strategies starts from 21-acetoxy-11β,17α-dihydroxy-5α-pregnan-3,20-dione, which undergoes dibromination by molecular bromine in acetic acid at positions C_2_ and C_4_, and then the resulting dibromide is dehydrobrominated by heating it in collidine, which gives prednisolone as an acetate at position C_21_. Hydrolyzing this compound leads to the formation of prednisolone [18] (Figure 3).

In order to overcome all the problems deriving from chemical synthesis, the potential of microbial steroid biotransformation has been known for several decades, as its application offers a number of advantages over chemical synthesis. These advantages concern (i) the regio- and/or stereospecific functionalization of molecules at positions not always available for chemical agents, (ii) how multiple consecutive reactions are carried out in a single operation step, and (iii) more ecofriendly processes (i.e., mild reaction conditions and aqueousmedia), as several studies have been conducted on the biotransformations of steroid compounds [19].

Recently the biotransformation of cortisone acetate and hydrocortisone acetate with *Arthrobacter simplex* (*Pimelobacter simplex*) to the corresponding prednisone acetate [20,21,22] and prednisolone acetate [23] has been reported. Moreover, prednisolone is also obtained from hydrocortisone by biotransformation with *Arthrobacter simplex* [24] using recombinant 3-ketosteroid-Δ1-dehydrogenases [25] and as a hydrocortisone biometabolite from unicellular microalgal cultures [26]. Furthermore, recently the 11β-reduction of prednisone by biotransformation capacities were confirmed to produce prednisolone [27].

On the other hand, biotransformations are widely used to obtain new steroidal derivatives that can have some therapeutic advantages, such as higher activity, longer half-life, and reduced side effects. In this field, various microorganisms afford the 20β-hydroxy-prednisone from prednisone [28] and 20β-hydroxy-prednisolone from prednisolone [29,30]. 

The *Actinobacteria* have been widely studied and their biotransformation capacities confirmed in various types of transformations of steroid rings, such as the reduction of the C-C and C-O double bonds, oxidation of the hydroxyl (OH) group, dehydrogenation and hydroxylation, and in many cases also in the lateral-chain degradation processes or the total degradation of the steroid ring [31].

Rhodococci belong to the order of *Actinomycetales*, they are gram-positive, obligate aerobic, non-mobile, and non-sporulating bacteria that are usually found in soil and aquatic environments and are considered very important as they can be used industrially [32]. Several studies have shown that the species belonging to this genus are able to grow both in mesophilic conditions [33] and in psychrophilic conditions [34].

In particular, *Rhodococcus* include aerobic bacterial species important for their ability to biotransform a wide range of steroids via hydroxylation or Bayer–Villiger oxidation, while the *Corynebacterium* and *Nocardia* genera are known to provide dehydrogenation products [31].

Moreover, in previous works the biotransformations of various bile acids with *Rhodococcus* spp. have been reported and Δ1-dehydrogenation, the partial degradation of the C_17_-side chain together with new 9,10-secosteroids derivatives, has been achieved [35,36]. The highlighted ability of these microorganisms to hydroxylate and dehydrogenate the steroid ring system prompted us to test the capabilities of various *Rhodococcus* strains towards the biotransformation of cortisone and hydrocortisone.

The purpose of this work is to evaluate the biotransformative capacity of some bacteria belonging to the *Rhodococcus* genus using cortisone and hydrocortisone as substrates with the aim of isolating products with greater therapeutic activity than the starting compounds. In this study we present the results of a preliminary screening followed by the isolation and characterization of the products obtained from batch cultures of the microorganisms that tested positive for the screening (strains with capacity to transform the cortisone and/or hydrocortisone).

After an initial lab-scale screening where 13 representatives of the *Rhodococcus* genus were tested towards the biotransformation of cortisone and hydrocortisone, preparative cultures of the strains that were found to be positive to verify the biotransformation of the products and their yields were set up.

## 2. Results and Discussion

### 2.1. Rhodococcus Screening and Selection

Due to the numerous harmful and dangerous effects of the reagents used in the chemical synthesis of prednisone and prednisolone, research is increasingly turning towards greener approaches. The use of bromine in the chemical synthesis can cause an explosive risk as well as irritating and respiratory effects if you come into contact with this reagent, also as regards 3,5-lutidine there are particular risks as it is flammable corrosive and toxic. To overcome all these problems, the biotransformative approach using non-pathogenic microorganisms has proved to be of great interest for the mild reaction conditions and the water-based culture medium of the microorganisms. In previous studies it has been shown that there is much evidence of the capacity of *Rhodococcus* strains to biotransform steroidal substrates to have Δ1-dehydrogenation products. [35,36]. Starting from these results, 13 *Rhodococcus* strains in the biotransformations of cortisone and hydrocortisone were tested. Screening tests were carried out in order to select microrganisms able to biotransformate these substrates. In Table 1 results of this screening are reported with the relative retention factors (Rf) of the product obtained.

Some of the tested bacteria were able to biotransform the administered substrates in particular, regarding the biotransformations of cortisone, *R. globerulus, R. aetherivorans, R. coprophilus, R. ruber,* and *R. rhodochrous* DSM 43273 converted the starting substrate into a product having Rf equal to 0.55. The reactions conducted with *R. erythropholis* and *R. baikonurensis,* in addition to the product just mentioned, generated another compound with an Rf of 0.23. 

Biotransformation activity has not been verified with *R. opacus*, *R. fascians*, *Rhodococcus* sp. R312, *R. rhodochrous* NCIMB 11216, and *R. equi*.

In the biotransformation of cortisone *R. zopfii* exhibited a particular behavior leading to a complete degradation of the substrate without providing biotransformation products. In this regard, it is known that some microorganisms are able to use steroids as a carbon source, degrading the lateral chain and subsequently the steroid nucleus, reaching complete mineralization [36]. 

**Figure 4:**
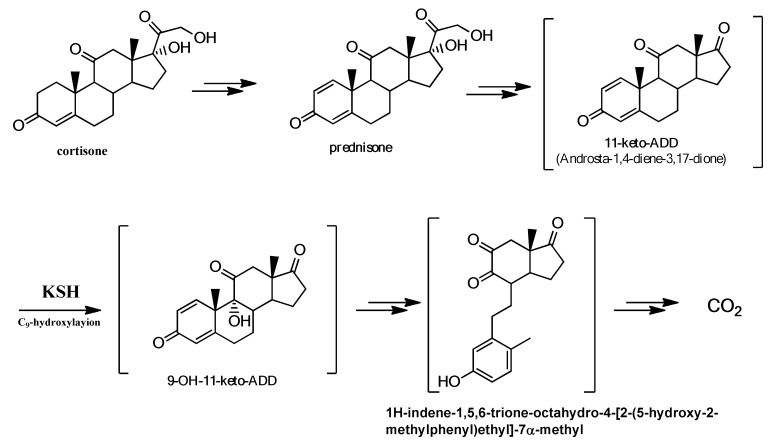
Metabolic pathway of steroid degradation.

Figure 4 shows the probable degradation pathway of cortisone by Actinobacteria [31] based on the literature data.

Steroids with 3-oxo-4-ene structure, such as cortisone and hydrocortisone, are subjected to the cleavage of ring B. The Δ1-dehydrogenation of cortisone to give prednisone is the initial stage that leads to the complete elimination of the C_17_ chain with the ADD (androstadienedione) formation. This is a classic step in the degradation of other steroids, such as sitosterols, testosterone, and bile acids [37].

ADD is subjected to hydroxylation at the C_9_-position catalyzed by 3-ketosteroid-9α-hydroxylase (KSH), a two-component iron-sulfur-containing monooxygenase [38,39,40]. This reaction leads to the spontaneous cleavage of the ring B by a retro-aldol cleavage and the formation of the aromatic 9,10-secosteroids, which undergo further degradation [41]. 

Because of this knowledge we can, therefore, assume that the Δ1-dehydrogenation is the initial stage that leads to the degradation of the C_17_-chain and to the opening of the ring B and subsequently to the total degradation of cortisone. Furthermore non-quantitative yields and the absence of collateral products and starting product confirm this assumption.

As for the biotransformations of hydrocortisone, in most cases a single product with an Rf of 0.45 was obtained, only in one case with *R. baikonurensis* a compound with an Rf of 0.21 was detected, while *R. erythropholis* was the only microorganism able to produce two compounds (Rf 0.45 and 0.21). *R. rhodochrous* DSM 43273 had completely metabolized the starting substrate without providing detectable products through thin-layer chromatography (TLC) analysis. Most likely, *R. rhodochrous* DSM 43273 completely degraded the carbon source following a metabolic pathway similar to that of cortisone as previously described. Finally, *R. opacus, R. fascians, R. aetherivorans, R. rhodochrous* NCIMB 11216, *Rhodococcus* sp. R312, and *R. equi* showed no biotransformative capacity towards hydrocortisone.

### 2.2. Characterization of the Biotransformation Products of Cortisone 

**Figure 5:**
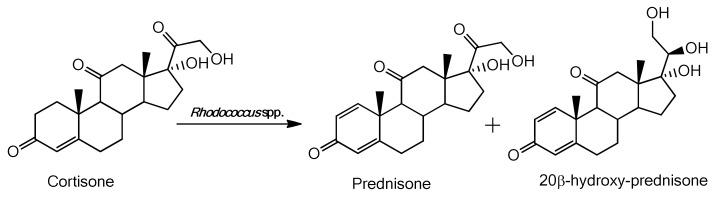
Products obtained from biotransformation of cortisone with *Rhodococcus* spp.

Semi-preparative cultures were necessary to isolate and identify the biotransformation products. The identification of the products was carried out by NMR analysis, comparing the signals obtained with those of the spectra present in the literature. In general, from the biotransformation of cortisone with *Rhodococcus* species, two products were obtained: prednisone and 20β-hydroxy-prednisone, the first being the product of the dehydrogenation of the C_1_-C_2_ bond in cortisone, while 20β-hydroxy-prednisone in addition to Δ^1^-dehydrogenation presents the reduction of the carbonyl group in the position C_20β_ as shown in Figure 5.

Table 2 shows the products of the biotransformations of cortisone with the relative yields and reaction times; the quantity of residual cortisone is also specified. The biotransformation yields were obtained by working out the percentage of the ratio between the moles of the product obtained compared to the moles of the administered substrate. The relative standard deviation value for statistical analysis was also reported.

The product of Δ^1^-dehydrogenation (prednisone) was the only one obtained from the biotransformations with *R. globerulus, R. aetherivorans, R. coprophilus, R. ruber,* and *R. rhodochrous* DSM 43273. Among these bacteria, only in one case (*R. coprophilus* after 24 h of biotransformation), was no residual cortisone observed, and the yield was very high (94%). This result clearly underlines this strategy as a valid alternative to the chemical synthesis of prednisone, since the time of biotransformation is rather short when whole cells are rather short, and the yield of the product is almost quantitative. Regarding the biotransformations of cortisone with *R. globerulus*, *R. aetherivorans*, and *R. ruber* after 72 h of incubation with the substrate, yields of prednisone production were quite similar: 33% with *R. globerulus*, and 35% and 52% with *R. aetherivorans* and *R. ruber,* respectively. In these cases, variable amounts of cortisone residue were observed (47–65%). The ^1^H- and ^13^C-NMR spectra showed the characteristic signals of the C_1_-C_2_ double bond at 6.19 (C_2_-H), 7.63 (C_1_-H), 127.7 (C_2_), and 158.4 (C_1_) ppm of prednisone. On the other hand, in biotransformations carried out with *R. baikonurensis* and *R. erythropolis*, after 72 h of reaction, in addition to prednisone with yields of 27% and 33% respectively, both bacteria produced a new compound corresponding to 20β-hydroxy-prednisone (yield 68% for *R. baikonurensis* and 37% for *R. erythropolis*). The stereochemistry of the C_20_-reduction is confirmed by comparing the ^1^H-NMR data (i.e., 4.61 (C_20_-H) and 4.52 (C_21_-H) ppm in C_5_D_5_N) and ^13^C-NMR data (i.e., 65.0 (C_21_) and 72.5 (C_20_) ppm) with data recorded for the commercially available compound. This product could be an interesting derivative of the bioactive prednisone.

### 2.3. Characterization of the Biotransformation Products of Hydrocortisone 

**Figure 6:**
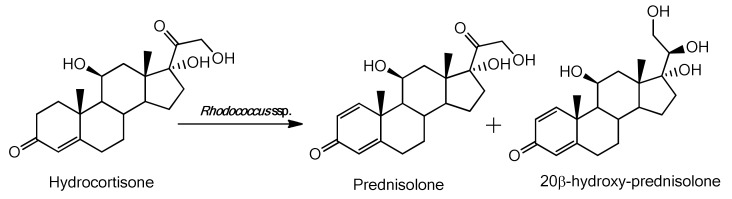
Products obtained from biotransformation of hydrocortisone with *Rhodococcus* spp.

As in the biotransformation of cortisone, in the case of hydrocortisone two types of compound were also obtained: prednisolone, deriving from the Δ^1^-dehydrogenation of the ring A, and 20β-hydroxy-prednisolone, which is a product of the reduction of the carbonyl group in the side chain (C_20_) as shown in Figure 6. 

The products obtained from the biotransformations of hydrocortisone with their relative yields and reaction times are reported in Table 3, and the quantities of residual hydrocortisone are also reported (where present). Yield percentages of biotrasformation have been calculated on the basis of the weights of the products obtained after chromatography separation. The relative standard deviation value for statistical analysis was also reported.

*R. coprophilus* is the only microrganism able to bioconvert hydrocortisone after 24 h in prednisolone with a yield equal to 97% without the presence of residual hydrocortisone; the ^1^H and ^13^C-NMR spectra show the characteristic signals 6.27 (C_2_-H), 7.24 (C_1_-H), 127.1 (C_2_) and 156.8 (C_1_) ppm of prednisolone. *R. globerulus* and *R. ruber* afforded the same product after 72 h of reaction with different yields (76% and 56%, respectively), but in these cases a certain amount of residual hydrocortisone was observed.

*R. baikonurensis* transformed hydrocortisone into 20β-hydroxy-prednisolone without recovering hydrocortisone. The stereochemistry of the C_20_-reduction is supported by the comparison of the recorded NMR data with the data given in the literature [29]: 3.85 (C_20_-H), 3.91 (C_21_-H), 65.3 (C_21_), and 79.1 (C_20_) ppm. The yield after 72 h of biotransformation is quite low (32%), and a possible explanation may be that the enzymes of the microorganism are able to form 20β-hydroxy-prednisolone and subsequently the substrate is metabolized via the steroid degradation pathway described previously. This process includes the initial oxidation of the ring A, degradation of the side chain, and finally complete mineralization.

Finally, *R. erythropolis* provided both the prednisolone and the 20β-hydroxy-prednisolone biotransformation products with yields of 37% and 40%, respectively, after a 72-hour-reaction. In this case, even using the maximum biotransformation time, 18% of residual hydrocortisone was recovered.

## 3. Materials and Methods 

### 3.1. Chemicals and Rhodococcus Strains

Cortisone and hydrocortisone were purchased from Merck KGaA (Darmstadt, Germany), and 20β-hydroxy-prednisone was purchased from Toronto Research Chemical Inc. (Toronto, Canada). Plate count broth (PCB) is commercially available from Oxoid (Basingstoke, UK) and contains tryptone enzymatic digest from casein 5 g/L, yeast extract 2.5 g/L, and glucose 1 g/L. All the other chemicals were purchased from Fluka Analytical (Steinheim, Germany) unless otherwise stated.

The 13 *Rhodococcus* strains belong to the collection of microorganisms of the Life Sciences and Biotechnology Department of the University of Ferrara and they were purchased from the DSMZ (Leibniz Institute DSMZ-German Collection of Microorganisms and Cell Cultures GmbH) company (Table 4). The master cell bank of every strain was maintained at −20 °C in cryovials in Plate count broth medium (1 mL) mixed with glycerol (0.5 mL) as a crioprotectant agent. The working cell bank was conserved at 4 °C in plate count agar (PCA) slants for 6 months and used for seed cultures. 

### 3.2. Rhodococcus Biotransformation Screening Conditions

For each of the selected *Rhodococcus* strains, a loopful of bacterial cells cultured on the plate count agar was transferred to 50 mL Erlenmeyer flasks containing 10 mL of sterile PCB medium and incubated at 30 °C and 110 rpm in an orbital shaker for 48 h. Each microorganism was inoculated in three Erlenmeyer flasks, closed with cotton plugs, and submitted to the same protocol.

After the bacteria were grown, a solution of the substrate (cortisone or hydrocortisone) in dimethylsulfoxide (DMSO) (0.1 mL containing 10 mg of substrate) was added. The course of the reactions was followed by withdrawing samples (1 mL) every 24 h up to 72 h from the administration of the substrate. The reactions were stopped after 72 h, as no significant differences were observed between the results of the TLC analysis performed at 48 h and 72 h. Samples were then centrifuged (6720 RCF; 10 min) in order to remove the cells, and the supernatants were extracted with ethyl acetate and immediately analyzed by TLC on silica gel using ethyl acetate as an eluent.

### 3.3. Semi-Preparative Biotransformation Protocol

The biotransformations showing additional spots other than the starting material were repeated on a semipreparative scale (200 mL) in order to isolate the products. For each of the selected *Rhodococcus* strains, a loopful of bacterial cells cultured on PCA was transferred to 50 mL Erlenmeyer flasks containing 20 mL of sterile PCB medium and incubated at 30 °C and 110 rpm in an orbital shaker for 48 h. The whole culture was introduced into a 500 mL Erlenmeyer flask containing 200 mL of sterile PCB, and after 48 h incubation at 30 °C and 110 rpm, the substrate (0.2 g) in DMSO (2 mL) was added and the culture was maintained in the same conditions for a maximum of 72 h. All tests were carried out in triplicate for statistical significance. A blank sample was also incubated containing all the reagents but no bacteria. The reaction course was monitored by withdrawing samples (1 mL) every 24 h up to 72 h of reaction, and the semipreparative reactions were followed by TLC analysis and stopped when the higher conversion was reached. TLC analysis was performed using 1 ml of sample taken every 24 h from the flask in which the reaction was conducted. The sample was subsequently subjected to centrifugation, extraction with ethyl acetate, and immediately analyzed using TLC on silica gel using ethyl acetate as an eluent, following the same protocol used in the screening procedure.

The cells were removed by centrifugation (5242 RCF, 20 min) and the supernatant was extracted with ethyl acetate (3 × 80 mL). The organic layer was dried over anhydrous Na_2_SO_4_, the solvent was evaporated, and the crude mixture was purified on a chromatographic column (silica gel, ethyl acetate/cyclohexane 50/50 as an eluent). The biotransformation yields were obtained by working out the percentage of the ratio between the moles of the product obtained compared to the moles of the administered substrate.

### 3.4. Analitical Methods

TLC was performed on precoated silica gel plates (thickness 0.25 mm, Merck) and silica gel (Fluka, Kiesegel 60, 70–230 mesh) was used for preparative column chromatography.

Melting points are uncorrected and were determined on a 510 Büchi melting point instrument. ^1^H and ^13^C NMR spectra were obtained with a Varian Gemini 300 spectrometer operating at 300 MHz (^1^H) and 100 MHz (^13^C), with Me_4_Si as an internal standard.

### 3.5. Compound Data

17α,21-dihydroxy-1,4-pregnadiene-3,11,20-trione (prednisone): m.p. 234 °C; ^1^H-NMR (CDCl_3_): δ 0.67 (s, 3H, C_18_-CH_3_); 1.42 (s, 3H, C_19_-CH_3_); 1.69 (m, 1H, C_7_-H); 1.73 (m, 1H, C_7_-H); 1.92 (m 1H; C_16_-H); 1.94 (m, 2H, C_15_-H); 1.98 (m, 1 H, C_8_-H); 1.98 (m, 1H, C_6_-H); 2.01 (m, 1H, C_6_-H); 1.99 (d, 1H, *J* = 3.1, C_9_-H); 2.06 (m, 1H, C_14_-H); 2.38 (m, 1H, C_12_-H); 2.72 (m 1H; C_16_-H); 2.77 (m, 1H, C_12_-H); 4.24 (d, 1H, *J* = 20 Hz, C_21_-H); 4.62 (d, 1H, *J* = 20 Hz, C_21_-H); 6.07 (t, 1H, *J* = 1.8 Hz, C_4_-H); 6.19 (dd, 1H, *J* = 10.2 and 1.8 Hz, C_2_-H); 7.63 (d, 1H, *J* = 10.2 Hz, C_1_-H); ^13^C-NMR (CDCl3): δ 16.2 (C_18_); 19.3 (C_19_); 24.1 (C_15_); 33.3 (C_7_); 34.9 (C_6_); 35.2 (C_16_); 37.4 (C_8_); 44.1 (C_10_); 50.8 (C_14_); 51.1 (C_12_); 52.3 (C_13_); 61.1 (C_9_); 67.8 (C_21_); 89.1 (C_17_); 124.7 (C_4_); 127.8 (C_2_); 158.2 (C_1_); 170.9 (C_5_); 188.6 (C_3_); 211.4 (C_11_); 212.7 (C_20_).

11α,17β,21-trihydroxy-1,4-pregnadiene-3,20-dione (prednisolone): m.p. 235 °C; ^1^H-NMR (CDCl_3_): δ 0.97 (s, 3H, C_18_-CH_3_); 1.08 (d, 1H, C_9_-H); 1.10 (m, 1H, C_7_-H); 1.45 (s, 3H, C_19_-CH_3_); 1.45 (m, 1H, C_15_-H); 1.55 (m, 1H, C_16_-H); 1.56 (m, 1H, C_12_-H); 1.71 (m, 1H, C_14_-H); 1.72 (m, 1H, C_15_-H); 2.09 (m, 1H, C_12_-H); 2.13 (m, 1H, C_7_-H); 2.15 (m, 1H, C_8_-H); 2.35 (m, 1H, C_6_-H); 2.57 (m, 1H, C_6_-H); 2.71 (m, 1H, C_16_-H); 4.28 (d, 2H, C_21_-H); 4.50 (s, 1H, C_11_-H); 6.02 (s, 1H, C_4_-H); 6.27 (d, 1H, C_2_-H); 7.24 (d, 1H, C_1_-H); ^13^C-NMR (CDCl_3_): δ 16.9 (C_18_); 20.9 (C_19_); 23.8 (C_15_); 31.2 (C_7_); 31.8 (C_6_); 33.5 (C_16_); 34 (C_8_); 38.9 (C_10_); 44.1(C_14_); 47.1 (C_12_); 51.1 (C_13_); 55.4 (C_9_); 66.5 (C_21_); 68.9 (C_11_); 88.6 (C_17_); 121.8 (C_4_); 127.1 (C_2_); 156.8 (C_1_); 170.5 (C_5_); 185.8 (C_3_); 212.1 (C_20_).

17α,20β,21-trihydroxy-1,4-pregnadiene-3,11-dione (20β-hydroxy-prednisone): m.p.240–242 °C; ^1^H-NMR (CDCl_3_): δ 0.81 (s, 3H, C_18_-CH_3_); 1.03 (m, 1H, C_15_-H); 1.43 (s, 3H, C_19_-CH_3_); 1.50 (m, 1H, C_7_-H); 1.77 (m, 1H, C_6_-H); 1.81 (m, 1H, C_7_-H); 1.82 (m, 1H, C_15_-H); 1.90 (m, 1H, C_14_-H); 1.92 (m, 1H, C_6_-H); 1.93 (m, 1H, C_8_-H); 2.01 (m, 1H, C_16_-H); 2.45 (m, 1H, C_9_-H); 2.48 (m, 1H, C_16_-H); 2.52 (m, 1H, C_12_-H); 2.56 (m, 1H, C_12_-H); 2.70 (m, 1H, C_12_-H); 3.76 (brs, 1H, C_20_-H) (4.61, m in C_5_D_5_N); 3.76 (brs, 1H, C_21_-H) (4.52, m in C_5_D_5_N); 6.06 (t, 1H, *J* = 1.26, C_4_-H); 6.18 (dd, 1H, *J* = 10.3, 1.9, C_2_-H); 7.67 (d, 1H, *J* = 10.2, C_1_-H); ^13^C-NMR (CDCl_3_): δ 15.9 (C_18_); 19.3 (C_19_); 24.5 (C_15_); 34.8 (C_7_); 35.0 (C_6_); 33.4 (C_16_); 37.7 (C_8_); 44.1 (C_10_); 49.5 (C_14_); 52.7 (C_12_); 52.8 (C_13_); 61.3 (C_9_); 65.0 (C_21_); 72.5 (C_20_); 85.0 (C_17_); 124.6 (C_4_); 127.7 (C_2_); 158.4 (C_1_); 171.2 (C_5_); 188.7 (C_3_); 213.3 (C_11_). 

11β,17α,20β,21-tetrahydroxy-1,4-pregnadiene-3-one (20β-hydroxy-prednisolone): mp 185–187 °C; ^1^H-NMR (CDCl_3_): δ 1.06 (s, 3H, C_18_-CH_3_); 1.11 (m, 1H, C_7_-H); 1.12 (d, *J* = 10, 1H, C_9_-H); 1.44 (s, 3H, C_19_-CH_3_); 1.52 (m, 1H, C_15_-H); 1.54 (m, 1H, C_16_-H); 1.6 (m, 1H, C_12_-H); 1.77 (m, 1H, C_14_-H); 1.8 (m, 1H, C_15_-H); 2.07 (m, 1H, C_12_-H); 2.12 (m, 1H, C_7_-H); 2.18 (m, 1H, C_8_-H); 2.23 (m, 1H, C_6_-H); 2.35 (m, 1H, C_6_-H); 2.45 (m, 1H, C_16_-H); 3.85 (m, 1H, C_20_-H); 3.91 (m, 2H, C_21_-H); 4.41 (s, 1H, C_11_-H); 6.02 (s, 1H, C_4_-H); 6.27 (dd, *J* = 10 and 1.5, 1H, C_2_-H); 7.29 (d, *J* = 10, 1H, C_1_-H); ^13^C-NMR (CDCl_3_): δ 16.39 (C_18_); 21.1 (C_19_); 23.2 (C_15_); 33.5 (C_7_); 31.9 (C_6_); 35.3 (C_16_); 33.2 (C_8_); 42.2 (C_10_); 55.1 (C_14_); 47.4 (C_12_); 46.9 (C_13_); 55.5 (C_9_); 65.3 (C_21_); 69.1 (C_11_); 79.1 (C_20_); 84.9 (C_17_); 120.9 (C_4_); 126.1 (C_2_); 159.2 (C_1_); 173.8 (C_5_); 187.8 (C_3_). 

## 4. Conclusions

In this work the ability of some strains belonging to the *Rhodococcus* genus to biotransform cortisone and hydrocortisone was tested. The aim of this work was to find new active compounds and alternative methods to synthesize chemical products already known. To reach this goal, we performed a screening of several *Rhodococcus* strains as it has been shown in the literature that they have a high ability to biotransform many substances including steroids. Lab-scale experiments allowed us to identify the positive strains (strains with the capacity to transform the cortisone and/or hydrocortisone), and subsequently, the preparation on a semi-preparative scale of the same allowed for the characterization of the products obtained with the relative biotransformation yields. For both substrates used, Δ^1^-dehydrogenation products (e.g., prednisone and prednisolone) were obtained, in particular with *R. coprophilus* where the bioconversion yields were practically quantitative, making it a good candidate for the synthesis of prednisone and prednisolone using a method that follows the philosophy of green chemistry.

With regard to the reduction products at the level of the carbonyl group in C_20_, the stereochemistry of the hydroxyl group was carried out by comparing NMR spectra of known substances or commercial standards.

In these biotransformations, this type of product has been obtained with lower yields than those previously described. If of interest, it could be useful to study the reaction conditions in order to optimize the yield of generation of these compounds.

## Figures and Tables

**Table 1:** Analitical results of *Rhodococcus* screening in biotransformation of cortisone and hydrocortisone.

Bacteria	Products from Cortisone	Products from Hydrocortisone
*R. zopfii*	Degradation	Rf 0.45
*R. opacus*	no product	no product
*R. fascians*	no product	no product
*R. baikonurensis*	Rf 0.23, 0.55	Rf 0.21
*R. globerulus*	Rf 0.55	Rf 0.45
*R. aetherivorans*	Rf 0.55	no product
*R. coprophilus*	Rf 0.55	Rf 0.45
*R. rhodochrous* NCIMB 11216	no product	no product
*Rhodococcus* sp. R312	no product	no product
*R. equi*	no product	no product
*R. ruber*	Rf 0.55	Rf 0.45
*R. rhodochrous* DSM 43273	Rf 0.55	degradation
*R. erythropholis*	Rf 0.23, 0.55	Rf 0.45, 0.21

**Table 2:** Biotransformations of cortisone with *Rhodococcus* spp.

Bacteria	Time of Biotransformation (h)	Prednisone (% Yield)	20β-Hydroxy-Prednisone (% Yield)	Cortisone Residue (%)
*R. baikonurensis*	72	27 ± 3 (0.053 ± 0.005 g )	68 ± 4 (0.134 ± 0.007 g)	--
*R. globerulus*	72	33 ± 6 (0.065 ± 0.012 g)	--	65 ± 3 (0.129 ± 0.006 g)
*R. aetherivorans*	72	35 ± 3 (0.069 ± 0.006 g)	--	63 ± 7 (0.125 ± 0.014 g)
*R. coprophilus*	24	94 ± 2 (0.185 ± 0.004 g)	--	--
*R. ruber*	72	52 ± 4 (0.102 ± 0.008 g)	--	47 ± 4 (0.093 ± 0.008 g)
*R. rhodochrous* DSM 43273	72	31 ± 3 (0.0065 ± 0.006)		53 ± 3 (0.105 ± 0.006)
*R. erythropolis*	72	33 ± 5 (0.065 ± 0.01 g)	37 ± 6 (0.073 ± 0.011 g)	28 ± 6 (0.056 ± 0.012 g)

**Table 3:** Biotransformations of hydrocortisone with *Rhodococcus* spp.

Bacteria	Time of Biotransformation (h)	Prednisolone (% Yield)	20β-Hydroxy-Prednisolone (% Yield)	Hydrocortisone Residue (%)
*R. baikonurensis*	72	--	32 ± 3 (0.063 ± 0.006 g)	--
*R. globerulus*	72	76 ± 2 (0.151 ± 0.004 g)	--	23 ± 1 (0.045 ± 0.002 g)
*R. coprophilus*	24	97 ± 2 (0.192 ± 0.004 g)	--	--
*R. ruber*	72	56 ± 4 (0.111 ± 0.009 g)	--	40 ± 4 (0.072 ± 0.007 g)
*R. erythropolis*	72	37 ± 5 (0.073 ± 0.01 g)	40 ± 8 (0.079 ± 0.016 g)	18 ± 6 (0.035 ± 0.012 g)

**Table 4:** *Rhodococcus* strains used for biotransformation screening of cortisone and hydrocortisone.

#	Genus	Species	Identification Code
1	*Rhodococcus*	*zopfii*	DSM 44108
2	*Rhodococcus*	*opacus*	DSM 43250
3	*Rhodococcus*	*fascians*	DSM 43985
4	*Rhodococcus*	*baikonurensis*	DSM 44587
5	*Rhodococcus*	*globerulus*	DSM 43954
6	*Rhodococcus*	*aetherivorans*	DSM 44752
7	*Rhodococcus*	*coprophilus*	DSM 43347
8	*Rhodococcus*	*rhodochrous*	NCIMB 11216
9	*Rhodococcus*	sp. *R312*	CBS 717.73
10	*Rhodococcus*	*equi*	IFO 3730
11	*Rhodococcus*	*ruber*	DSM 6264
12	*Rhodococcus*	*rhodochrous*	DSM 43273
13	*Rhodococcus*	*erythropholis*	DSM 43188

## References

[1] Fernandes P., Cabral J.M.S. (2007). Phytosterols: Applications and recovery methods. Bioresour. Technol..

[2] Hannich J.T., Umebayashi K., Riezman H. (2011). Additional Perspectives on The Biology of Lipids. Cold Spring Harbour Perspect. Biol..

[3] Muthukrishnan S., Merzendorfer H., Arakane Y., Kramer K.J., Gilbert L.I. (2012). Chitin Metabolism in Insects. Insect Molecular Biology and Biochemistry.

[4] Tong W.Y., Dong X. (2009). Microbial Biotransformation: Recent Developments on Steroid Drugs. Recent Pat. Biotechnol..

[5] Fragkaki A.G., Angelis Y.S., Koupparis M., Tsantili-Kakoulidou A., Kokotos G., Georgakopoulos C. (2009). Structural characteristics of anabolic androgenic steroids contributing to binding to the androgen receptor and to their anabolic and androgenic activities. Applied modifications in the steroidal structure. Steroids.

[6] Agoston E.S., Hatcher M.A., Kensler T.W., Posner G.H. (2006). Vitamin D Analogs as Anti-Carcinogenic Agents. Anti. Cancer Agents Med. Chem..

[7] Tuba Z., Bardin C.W., Dancsi A., Francsics–Czinege E., Molnár C., Csörgei J., Falkay G., Samuel S. (2000). Synthesis and biological activity of a new progestogen, 16-methylene- 17α-hydroxy-18-methyl-19-norpregn-4-ene-3,20-dione acetate. Steroids.

[8] Díaz-Chico N., Germán Rodríguez F., González A., Ramírez R., Bilbao C., Cabrera de León A., Aguirre Jaime A., Chirino R., Navarro D., Díaz-Chico J.C. (2007). Androgens and androgen receptors in breast cancer. J.Steroid Biochem..

[9] Chrousos G.P. (2015). Basic & Clinical Pharmacology. Adrenocorticosteroids & Adrenocortical Antagonists.

[10] Craigie E., Mullins J.J., Bailey M.A., Bader M. (2009). Glucocorticoids and mineralocorticoids. Cardiovascular Hormone Systems: From Molecular Mechanisms to Novel Therapeutics.

[11] Chung S.K., Ryooa C.H., Yang H.W., Shim J.-Y., Kang M.G., Lee K.W., Kang H.I. (1998). Synthesis and bioactivities of steroid derivatives as antifungal agents. Tetrahedron.

[12] Suzuki K., Nakata T., Shimizu T. (1998). Anti-Obesity Agents Patent.

[13] Dombrowski A.W., Hazuda D.J., Polishook J.D., Felock P.J., Singh S.B., Zink D.L. (2000). HIV-Integrase-Inhibitors. World Patent.

[14] Arthan D., Svasti J., Kittakoop P., Pittayakhachonwut D., Tanticharoen M., Thebtaranonth Y. (2002). Antiviral isoflavonoid sulfate and steroidal glycosides from the fruits of Solanum torvum. Phytochemistry.

[15] Funder J.W. (2010). Minireview: Aldosterone and mineralocorticoid receptors: Past, present, and future. Endocrinology.

[16] Zhang H., Tian Y., Wang J., Li Y., Wang H., Mao S., Liu X., Wang C., Bie S., Lu F. (2013). Construction of engineered *Arthrobacter simplex* with improved performance for cortisone acetate biotransformation. Appl. Microbiol. Biotechnol..

[17] Samuel S., Nguyen T., Choi H.A. (2017). Pharmacologic Characteristics of Corticosteroids. J. Neurocritical Care.

[18] Olivetq E.P., Gould D.H. (1959). US Patent.

[19] Fernández-Cabezón L., Galán B., García J. (2018). New Insights on Steroid Biotechnology. Front Microbiol..

[20] Mao S., Yu L., Ji S., Liu X., Lu F. (2016). Evaluation of deep eutectic solvents as co-solvent for steroids 1-en-dehydrogenation biotransformation by *Arthrobacter simplex*. J. Chem. Technol. Biotechnol..

[21] Gao Q., Shen Y.B., Huang W., Wang M. (2015). Effect of Natural Cyclodextrins on Cell Growth, Activity and Permeability of *Arthrobacter simplex*. Appl. Mech. Mater..

[22] Luo J., Ning J., Wang Y., Cheng Y., Zheng Y., Shen Y., Wang M. (2014). The effect of ethanol on cell properties and steroid 1-en-dehydrogenation biotransformation of *Arthrobacter simplex*. Biotechnol. Appl. Biochem..

[23] Spassov G., Krützfeldt R., Sheldrick W.S., Wania W., Vlahov R., Snatzke G. (1983). Crystallographic monitoring of microbiological steroid transformations. Eur. J. Appl. Microbiol. Biotechnol..

[24] Vlahov R., Pramatarova V., Spassov G., Suchodolskaya G.V., Koshcheenko K.A. (1990). Transformation of microcrystalline hydrocortisone by free and immobilized cells of *Arthrobacter simplex*. Appl. Microbiol. Biotechnol..

[25] Wang X., Feng J., Zhang D., Wu Q., Zhu D., Ma Y. (2017). Characterization of new recombinant 3-ketosteroid-Δ1-dehydrogenases for the biotransformation of steroids. Appl. Microbiol. Biotechnol..

[26] Ghasemi Y., Rasoul-Amini S., Morowvat M.H., Raee M.J., Ghoshoon M.B., Nouri F., Negintaji N., Parvizi R., Mosavi-Azam S.B. (2008). Characterization of Hydrocortisone Biometabolites and 18S rRNA Gene in *Chlamydomonas reinhardtii* Cultures. Molecules.

[27] Bie S., Lu F., Liu X., Mao S., Li J. (2012). Method for Manufacturing Prednisolone with Biotransformation. Patent CN 2012.

[28] Choudhary M.I., Siddiqui Z.A., Musharraf S.G., Nawaz S.A. (2005). Microbial transformation of prednisone. Nat. Prod. Res..

[29] Zhang W., Cui L., Wu M. (2011). Transformation of prednisolone to a 20β-hydroxy-prednisolone compound by *Streptomyces roseochromogenes* TS79. Appl. Microbiol. Biotechnol..

[30] Mohamed S.S., El-Hadi A.A. (2017). Biotransformation of prednisolone to hydroxyl derivatives by *Penicillium aurantium*. Biocatal. Biotransformation.

[31] Donova M.V. (2007). Transformation of steroids by actinobacteria: A review. Appl. Biochem. Micro..

[32] Krivoruchko A., Kuyukina M., Ivshina I. (2019). Advanced *Rhodococcus* Biocatalysts for Environmental Biotechnologies. Catalysts.

[33] Lichtinger T., Reiss G., Benz R. (2000). Biochemical identification and biophysical characterization of a channel-forming protein from *Rhodococcus erythropolis*. J. Bacteriol..

[34] Patrauchan M.A., Florizone C., Dosanjh M., Mohn W.W., Davies J., Eltis L.D. (2005). Catabolism of benzoate and phthalate in *Rhodococcus* sp. strain RHA1: Redundancies and convergence. J. Bacteriol..

[35] Costa S., Giovannini P.P., Fantin G., Medici A., Pedrini P. (2013). New 9,10-secosteroids from biotransformations of bile acids with *Rhodococcus ruber*. Helv. Chim. Acta.

[36] Costa S., Giovannini P.P., Fantin G., Medici A., Pedrini P. (2013). New 9,10-secosteroids from biotransformations of hyodeoxycholic acid with *Rhodococcus spp*. Helv. Chim. Acta.

[37] Horinouchi M., Hayashi T., Kudo T. (2012). Steroid degradation in *Comamonas testosteroni*. J. Steroid Biochem. Mol. Biol..

[38] Hu Y., Van Der Geize R., Besra G.S., Gurcha S.S., Liu A., Rohde M., Singh M., Coates A. (2010). 3-Ketosteroid 9α-hydroxylase is an essential factor in the pathogenesis of *Mycobacterium tuberculosis*. Mol. Microbiol..

[39] Capyk J.K., D’Angelo I., Strynadka N.C., Eltis L.D. (2009). Characterization of 3-ketosteroid 9α-hydroxylase, a Rieske oxygenase in the cholesterol degradation pathway of Mycobacterium tuberculosis. J. Biol. Chem..

[40] Petrusma M., Dijkhuizen L., Van Der Geize R. (2009). *Rhodococcus rhodochrous* DSM 43269 3-ketosteroid 9α-hydroxylase, a two-component iron-sulfur-containing monooxygenase with subtle steroid substrate specificity. Appl. Environ. Microbiol..

[41] Park R.J. (1984). Phenolic 9,10-secosteroids as products of the catabolism of bile acids by a Pseudomonas sp.. Steroids.

